# Blocking the Metabolic Switch Toward Cytosolic 1C Flux: A Novel Therapeutic Approach for Tumors With Low SLC19A1 Expression

**DOI:** 10.3389/pore.2022.1610337

**Published:** 2022-04-22

**Authors:** Zhe Chen, Hong Zhou, Haoliang Hu, Linxi Chen

**Affiliations:** ^1^ Institute of Pharmacy and Pharmacology, Hunan Provincial Key Laboratory of Tumor Microenvironment Responsive Drug Research, College of Basic Medical Science, Hengyang Medical School, University of South China, Hengyang, China; ^2^ Radiology Department, The First Affiliated Hospital of University of South China, Hengyang, China; ^3^ Changde Research Centre for Artificial Intelligence and Biomedicine, College of Life and Environmental Sciences, Hunan University of Arts and Science, Changde, China

**Keywords:** One-carbon units, SHMT1, SHMT2, RPMI, TSH, MTHFD1

To the Editor

One carbon (1C) unit metabolism plays a central role in supporting cell growth and proliferation in the body through the perception of cell glucose, amino acid and other nutritional statuses. The 1C metabolism pathway provides cellular components, including nucleotides, lipids, and proteins, for cell growth *via* folic acid and methionine cycles. The 1C metabolism pathway also generates glutathione and S-adenosylmethionine to maintain cellular redox status and epigenetic status with cytosolic or mitochondrial folate metabolism. Targeting 1C unit metabolic enzymes and downstream nucleic acid metabolic enzymes is seen as a viable cancer treatment method since the metabolism of amino acids (such as serine and glycine) and the 1C units they provide are sufficient to promote tumor growth [1].

Mitochondrial 1C flux is consistently overexpressed in cancer and supplied by the folic acid cycle and the mitochondrial serine-hydroxymethyltransferase (SHMT2), while cytosolic 1C flux is induced by the cytosolic enzyme SHMT1. Mitochondrial and cytosolic 1C flux is responsible for DNA replication and methylation. However, several studies have indicated that blocking mitochondrial 1C flux with SHMT2 inhibitors cannot abolish the neoplasia of hepatocellular cancer cells absolutely, whereas targeting mitochondrial and cytosolic 1C flux with dual SHMT1/2 inhibitors can restrain the formation of transplanted tumors completely. Hence, it is hinted that cytosolic 1C flux may also play an unexpected role in tumor progression under some nutrient availability.

Lee et al. [2] identified that tumor reliance on cytosolic versus mitochondrial 1C flux depends on folate levels, thereby challenging the current opinion that mitochondrial 1C flux is the sole contributor of 1C units in tumors. To demonstrate that 1C flux is determined by folic acid levels in cancer cells, the author analyzed cytosolic versus mitochondrial 1C metabolic flux in various tumors under normal physiological folate concentration. The results showed that SHMT1-mediated cytosolic 1C metabolic flux is the primary source of 1C units in a series of cancers, including T cell acute lymphoblastic leukemia, glioblastoma, and non-small-cell lung carcinoma under normal physiological folate concentration, whereas mitochondrial 1C flux is overly repressed, suggesting that tumor-specific reliance on cytosolic 1C flux depends on poor folate availability. In this study, the 1C metabolic flux of cancer cells is switched primarily toward the cytosolic folate cycle through SHMT1 to face nutrient availability under normal physiological folate concentration. Furthermore, the increased reliance on SHMT1 for producing 1C units is followed by a decrease in whole-cell SHMT flux, indicating a prominent drop specifically in SHMT2 flux under normal physiological folate concentration. It has been identified that cytoplasmic SHMT1 is overexpressed in lung cancer. SHMT1-dependent apoptosis is due to an increased accumulation of uracil during DNA replication [3]. In proliferating cancer cells, cytosolic 1C pathway flux through SHMT1 is essential to compensate for DNA replication and maintain tumor growth under nutrient-poor conditions. Notably, metabolic switch generally exists in tumor cells under poor environmental conditions. Cancer cells can switch their metabolic phenotypes to adapt to changes in their surroundings [4]. For example, brain tumor cells accommodate deprivation and preserve their survival by upregulating glucose transporters and switching to a more glycolytic phenotype. The metabolic switch helps breast cancer cells survive under hypoxia. Human high-grade glioma cancer cells also undergo metabolic shifts in response to the chemotherapy environment and induce therapy resistance. More importantly, metabolic switch inhibition prominently alleviates tumor progression, suggesting that targeting metabolic switch can be a promising strategy for tumor treatment [2].

Mechanically, 1C metabolic switch toward tumor-specific cytosolic 1C flux is mainly attributed to the decreased expression of SLC19A1 that encodes the reduced folate carrier (RFC). Low RFC expression causes the poor capacity to retain intracellular folates in cancer cells, thereby resulting in SHMT1-mediated reliance on cytosolic 1C metabolic flux. Furthermore, silencing SHMT1 with low SLC19A1 expression inhibits tumor growth by impairing pyrimidine biosynthesis *in vitro* and *in vivo*, whereas silencing SHMT1 with SLC19A1 overexpression cannot alleviate tumor growth. Consequently, a decrease in SLC19A1 makes tumors susceptible to SHMT1 and determines the 1C metabolic switch toward cytosolic 1C metabolic flux under physiological folate conditions. Inhibiting SHMT1 offers potential therapeutic options for tumor patients with low SLC19A1 expression.

In the future, SLC19A1 may be a marker for tumor patients that have an increased reliance on cytosolic 1C metabolic flux. Inhibiting SHMT1 expression may be an emerging strategy for tumor patients with low SLC19A1 expression (Figure 1). Currently, most drugs that selectively target SHMT2, such as antifolates lometrexol and pemetrexed, are recognized as anti-cancer drugs with long-term activity. However, the potential function of SHMT1 inhibitors has always been concerning for treating tumor diseases (Table 1). For example, compound 2.12, a derivative of the pyrazolopyran scaffold, has a lower dissociation constant when binding to the SHMT1 enzyme-serine complex and is preferred to inhibit SHMT1, eventually leading to cell death in lung cancer cells [5]. Mimosine, a naturally occurring plant amino acid, can chelate zinc, thereby inhibiting the SHMT1 promoter and blocking the its transcription [6]. Arsenic trioxide can increase the ubiquitin degradation of methylenetetrahydrofolate dehydrogenase 1 (MTHFD1) and SHMT1 to inhibit nuclear *de novo* thymidylate (dTMP) biosynthesis, thus increasing genome instability [7]. The spiro-dihydroindene analogs with a spirocyclic scaffold potently inhibit human cytosolic and plasmodial SHMT1 [8]. Tetrahydrofolate (THF) decreases SHMT1 production through the pH dependent-substrate inhibition [9]. 3-bromopyruvate (3BP) can bind to the active site of SHMT1 (Cys204) to form the enzyme-3BP complex, which completely inhibits human SHMT1 to achieve an anti-tumor effect [10]. The SHMT1-targeted inhibitor AGF347, a novel pyrrolo [3,2-d] pyrimidine compound, shows anti-tumor efficacy in HCT116 colon cancer, H460 non-small cell lung cancer (NSCLC), and MIA PaCa-2 pancreatic cancer [11]. However, AGF347 is not selective for SHMT1 and SHMT2. Besides, antifolate compounds are used clinically for treating cancers by inhibiting SHMT1. Lometrexol is an SHMT2 inhibitor and one of the best SHMT1 inhibitors in a panel of antifolate drugs [12]. MicroRNA-198 can downregulate SHMT1 expression to suppress the proliferation of lung adenocarcinoma cells, which shows a more effective alteration than silencing SHMT1 with siRNA [13]. Notably, microRNA-218-5p inhibits SHMT1 to suppress the natural killer (NK) cells and exerts a negative effect on lung adenocarcinoma [14].

**FIGURE 1:**
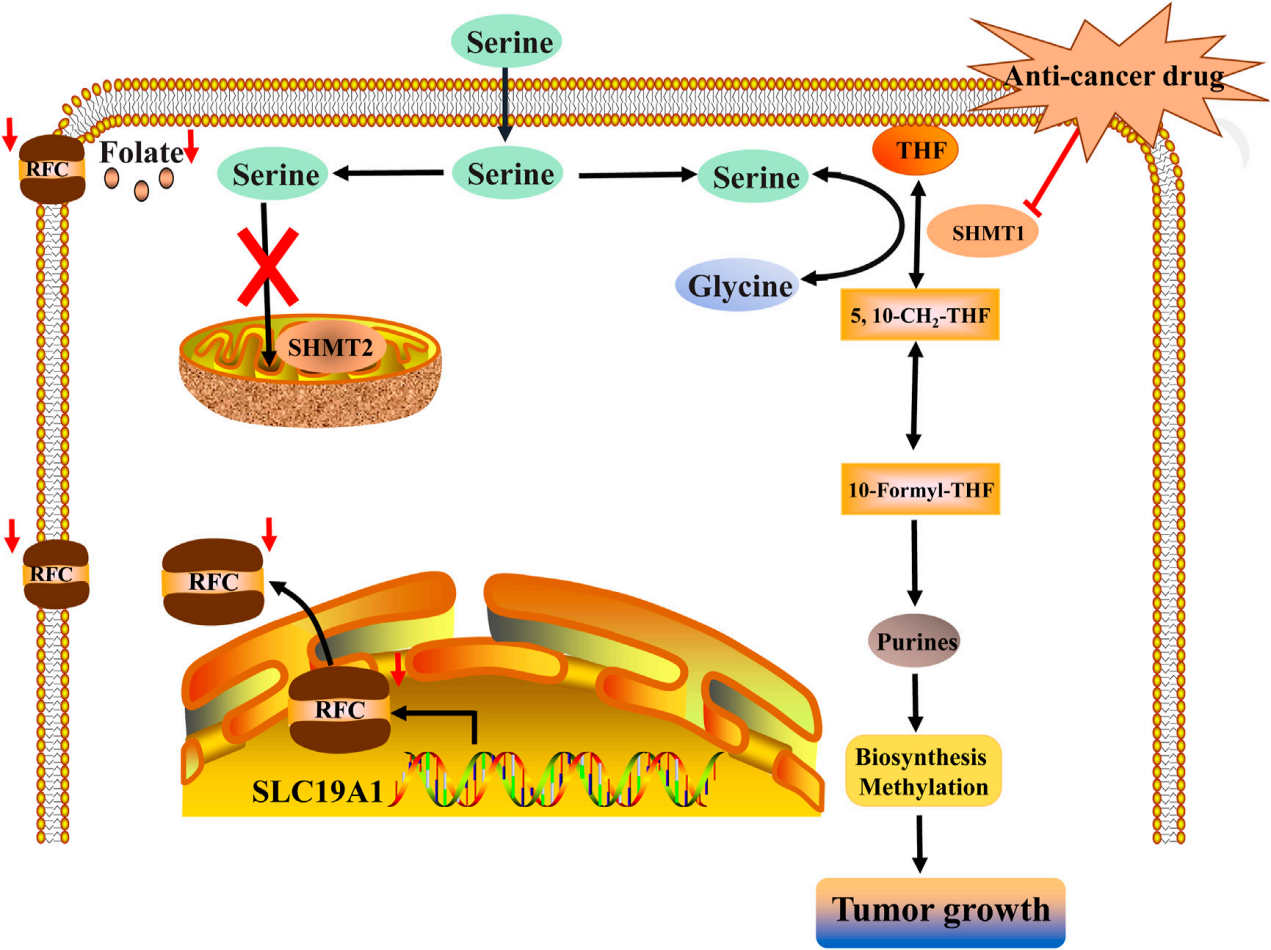
Inhibiting SHMT1 may be a vital target for a series of tumor diseases with low SLC19A1 expression. Low expression of SLC19A1 causes the poor capacity of cancer cells to retain intracellular folates, thereby resulting in a low level of folate conditions. Then, a 1C metabolic switch toward cytosolic 1C flux rather than mitochondrial 1C flux enables cancer cells to depend on SHMT1. Finally, SHMT-mediated cytosolic 1C metabolic flux can provide DNA replication and methylation for the growth of tumors with low SLC19A1 expression. Inhibiting SHMT1 can impair the growth of tumors with low SLC19A1 expression.

**TABLE 1:** The analysis of potential SHMT1 inhibitors.

Name	Structure	Mechanisms	Effects	Refers
Pyrazolopyran scaffold derivative 2.12	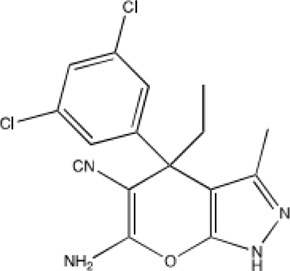	Lower dissociation constant by 50-fold	Causing apoptosis in lung cancer cell lines	[5]
Mimosine	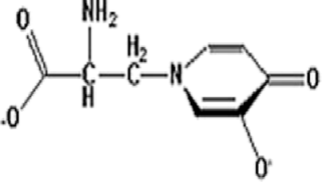	Inhibit transcription by chelating zinc	Inhibiting DNA replication	[6]
Arsenic trioxide	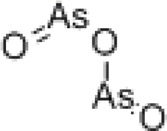	SHMT1 degradation	Increasing genome instability	[7]
Spiro-dihydroindene analogues	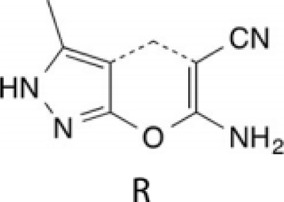	Strong target affinities	Inhibiting plasmodial DNA replication	[8]
Tetrahydrofolate	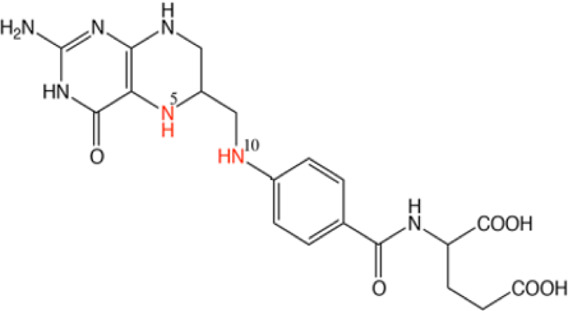	Substrate inhibition dependent on pH	Adapting cellular environments	[9]
3-bromopyruvate	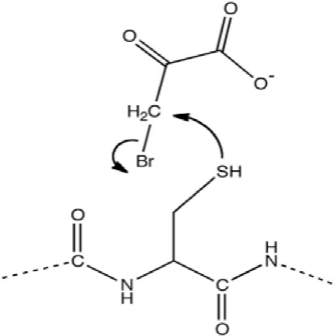	Reacting with Cysteine residues in a nucleophilic substitution	As a potent novel anti-tumour agent	[10]
AGF347	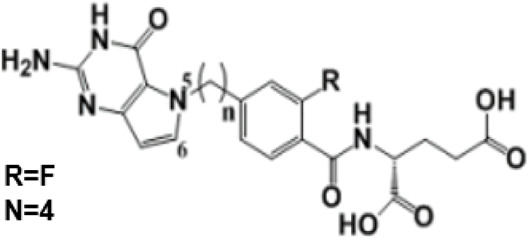	Molecular Modeling to design small-molecule inhibitor	Showing antitumor efficacies against lung, colon, and pancreatic cancer	[11]
Lometrexol	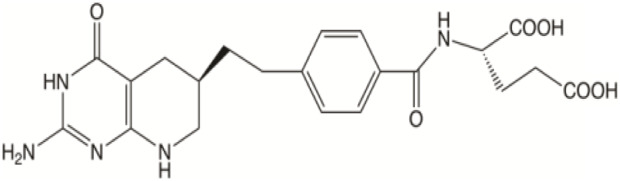	Targeting GARFT	As panel antifolates to treat cancer	[12]
miR-198	3′CTT​GGA​TAG​AGG​GGA​GAC​CTG	Binding to 3′ UTR1 of SHMT1 mRNA to downregulating its expression	Suppressing lung adenocarcinoma cells proliferation	[13]
MiR-218-5p	3′UGU​ACC​AAU​CUA​GUU​CGU​GUU-5′	Binding to 3′ UTR1 of SHMT1 mRNA to downregulating its expression	Suppressing killing effect of NK cells to lung adenocarcinoma	[14]

This paper reveals the diversity of 1C metabolism under different conditions in cancer cells. To better accommodate the poor environment, cancer cells are forced to adopt metabolic switch to maintain their growth and development. Selective inhibition of the specific isoform of SHMT may be a key target for many human tumors with metabolic switch. Nevertheless, the relationship between SHMT1 and SHMT2 in human tumors is extremely complicated. It has been demonstrated that SHMT1 can utilize its RNA-binding function to bind the 5′untranslated region of the SHMT2 transcript (UTR2) and control the function of SHMT2-expressed cancer cells. Inversely, the transfection of UTR2 in cancer cells can also modulate SHMT1 activity and reduce cell viability, suggesting that it is of great necessity for us to give more studies on the relationship between SHMT1 and SHMT2. Going forward, it needs to be carefully evaluated how to better utilize 1C metabolism to target tumors.

## Abbreviations

1C units, One-carbon units; DMEM, Dulbecco’s modified eagle medium; FBP1, Fructose-1,6-biphosphatase; MTHFD1, Methylenetetrahydrofolate dehydrogenase 1; RFC, The reduced folate carrier; RPMI, Roswell Park Memorial Institute; SHMT1, Cytosolic serine-hydroxymethyltransferase; SHMT2, Mitochondrial serine-hydroxymethyltransferase; TSH, Tetrahydrofolate; UTR2, the 5′untranslated region of the SHMT2 transcript.
